# A bibliometric analysis of global forest ecology research during 2002–2011

**DOI:** 10.1186/2193-1801-2-204

**Published:** 2013-05-02

**Authors:** Yajun Song, Tianzhong Zhao

**Affiliations:** School of Information Science & Technology, Beijing Forestry University, No.35 Tsinghua East Road, Beijing, Haidian District, 100083 P.R. China; Library of Beijing International Studies University, No.1 Dingfuzhuang Nanli, Beijing, Chaoyang District, 100024 P.R. China

**Keywords:** Article analysis, Bibliometric, Forest ecology, Java, Keyword frequency analysis

## Abstract

Bibliometric is increasingly used for the analysis of discipline dynamics and management related decision-making. This study analyzes 937,923 keywords from 78,986 articles concerning forest ecology and conducts a serial analysis of these articles’ characteristics. The articles’ records, published between 2002 and 2011, were downloaded from the Web of Science, and their keywords were exported by Java processing programs. The result shows that forest ecology studies focused on forest diversity, conservation, dynamics and vegetation in the last decade. Developed countries, such as the USA, Canada, and Germany, were the most productive countries in the field of forest ecology research. From 2002 to 2011, the number of articles published annually related to forest ecology grew at a stable rate, as indicated by the fit produced by a high determination coefficient (R^2^ = 0.9955). The findings of this study may be applicable for planning and managing forest ecology research and partners involved in such research may use this study as a reference.

**Electronic supplementary material:**

The online version of this article (doi:10.1186/2193-1801-2-204) contains supplementary material, which is available to authorized users.

## Introduction

Bibliometric analysis is an important part of reference and research services. Forest ecology is closely related to forest management and many studies have been performed from various perspectives, including studies of ecosystems at multiple forest spatial scales ( Rodrigues et al. 2011 ; Sitzia et al. 2010 ), long term ecosystem change ( Diaz et al. 2007 ; van Oudenhoven et al. 2012 ), climate change ( Cheaib et al. 2012 ; Şekercioğlu et al. 2012 ), soils (McLachlan and Bazely 2003 ; Wang et al. 2011 ), physiography (Morrissey et al. 2009 ; Rubio and Escudero 2005 ), carbon balance (Mitchell et al. 2009 ; Sillett et al. 2010 ), nutrient cycling (Berger et al. 2009 ; XU and Chen 2006 ), landscape ecology (Loucks et al. 2001 ; Wintle et al. 2005 ) and biodiversity (Hanberry et al. 2012 ; Lamb et al. 2005 ). In addition to these studies, a bibliometric analysis of global forest ecology could provide a fresh look at the current status of global forest ecology research and help identify hot spots.

In recent years, along with its continuously expanding range of application, bibliometric analysis plays an increasingly important role in management and decision-making in science and technology. It has been used to document the development of some research fields (Grandjean et al. 2011 ; Hendrix 2008 ; Narotsky et al. 2012 ; van Eck et al. 2010 ; van Raan 2006 ), including forestry (Dobbertin and Nobis 2010 ; Perez et al. 2004 ).

In this study, we perform a bibliometric analysis of forest ecology research over the last 10 years (2002–2011) aimed at (1) examining the temporal hot topics of forest ecology research by keyword frequency analysis, (2) revealing the distribution of articles by country/region, organization, funding agency, research area, author, year and publication name for articles covering forest ecology research and revealing advancements in forest ecological research, and (3) providing a new keywords frequency analysis method, which may benefit future research.

## Materials and methodology

### Data collection

Literature records, our analytical objects, were derived from the Web of Science, an online academic citation index database provided by Thomson Reuters. To define search terms, we used the “thesaurus” tool of Commonwealth Agricultural Bureaux (CAB) Abstracts.

**Figure 1:**
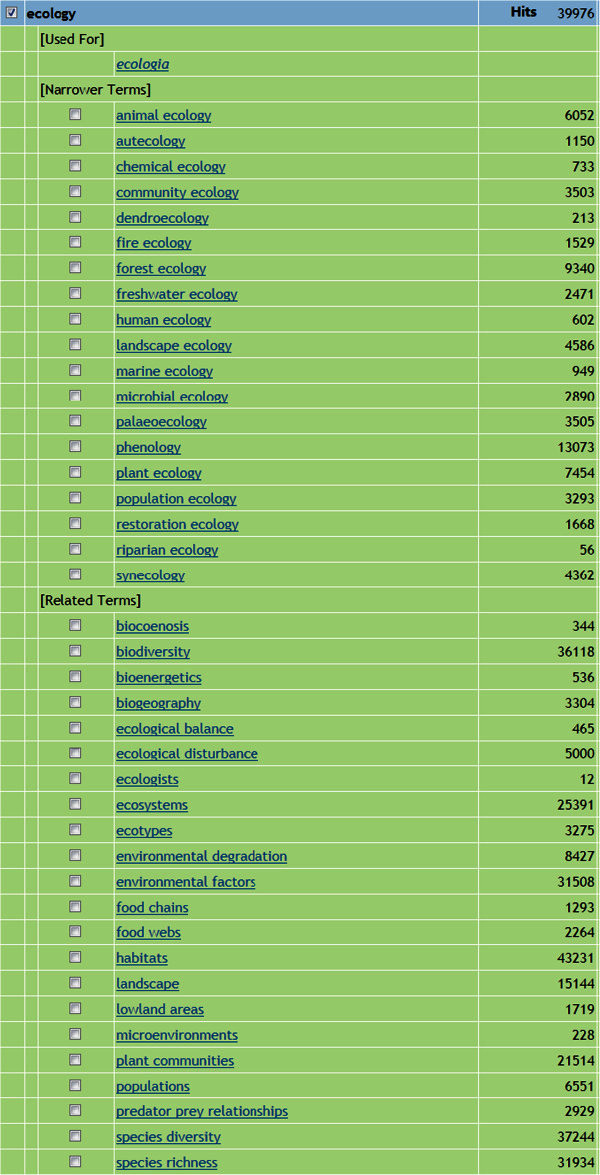
**Narrower terms and 22 related terms of ecology.**

We conducted a search on the word “ecology” in CAB Abstracts and the search produced 41 terms, including 19 narrower terms and 22 other related terms (Figure 1). We selected terms with more than 200 hits and used Microsoft Excel to rank them in descending order. We then removed the words “ecology” and “forest” from the Excel sheet and added the terms “climate,” “soils,” “physiography,” “carbon balance” and “nutrient cycling,” based on the concepts related to forest ecology defined by Barnes et al. ( 1997 ). Then, we defined the remaining 43 search terms and constructed a new search query. The search was limited to “article” type publications published between 1 January 2002 and 31 December 2011 in English.

The search query included 43 terms (see Appendix A). This query was run in Web of Science, which is a citation database of the Web of Knowledge, and a total of 78,986 forest ecology-related articles were identified.

Using the Web of Science’s analysis tools, we exported the 78,986 articles by country/region, organization, funding agency, research area, author, year, and publication. The statistical methods used by the Web of Science for the above statistical indicators of multi-author articles do not distinguish between the order of author’s locations, which may result the sum of these statistical result was greater than 78,986. The article records, including title, author, keywords, abstract, and organization, were exported in full record mode from the Web of Science to text files. A total of 158 text files were created, because the Web of Science limits each export to 500 records. In every text file, “author keywords” were marked by “DE,” and “keywords plus” were provided by the Web of Science and marked by “ID”. Both these two kinds of keywords were considered in this study.

### Keywords analysis

First, the frequency of each keyword was counted in each text file. We developed a java program named count.java (Additional file 1: Appendix B) using Eclipse software, a famous cross-platform integrated development environment. This java program can find and select keywords in the output text file by identifying parameters, and connect each keyword to a long string, while deleting the carriage returns. After detection, the keywords in the string were split by semicolons, and counted using HashMap traversal algorithm. The HashMap traversal result was saved to an array and sorted by the counters; then, the sorted result was exported to an intermediate file.

Second, the 158 intermediate files were merged, and the frequency of each keyword was counted. We developed a java program named merge.java (Additional file 1: Appendix C) using Eclipse software. When this program was run, the intermediate files defined in the input parameters were opened, and the keywords and their counters were saved to a HashMap. Then the keywords were counted again with HashMap traversal algorithm: the counters of the same keywords were added. Then, the HashMap traversal result was saved to an array, sorted by the counters, and exported into a result file.

Third, we developed a program (Additional file 1: Appendix D) to create a java package named frequency.jar to store the compiled java class files which were produced by compiling count.java and merge.java.

Fourth, we developed a batch program named count.bat (Additional file 1: Appendix E) to call the count.class with the input parameters “DE” and “ID”. All 158 text files were processed one by one. As a result, 158 intermediate files were created.

Fifth, we developed another batch program named merge.bat (Additional file 1: Appendix F) to call the merge.class with the input parameters, that is, the 158 intermediate files, to merge them. As a result, a final file was created, in which all keywords in 78,986 articles were counted and sorted.

After data processing, 937,923 keywords from those 78,986 articles were merged into 150,974 keywords. All of the keywords were sorted in reverse order based on their frequencies. The 100 most frequently used keywords became the focus of our study.

## Results

### Keywords analysis results

To narrow the research scope, the 100, 200, 300 most frequently used keywords were selected and analyzed. As a result, the 100 most frequently used keywords, 0.07% of the 150,974 unique keywords analyzed here represented 18.54% of the total (937,923) of all keywords harvested (Table 1). We focused on the top 100 keywords to examine the hot topics of forest ecology research (Table 2).

**Table 1:** **The top 100, 200, 300 keyword ratio and their frequencies**

Keywords number	Keywords ratio	Keywords frequencies	Frequencies Ratio (%)
100	0.07%(100/150974)	173925	18.54%(173925/937923)
200	0.13%(200/150974)	233042	24.85%(233042/937923)
300	0.20%(300/150974)	271233	28.92%(271233/937923)

**Table 2:** **The top 100 keywords in forest ecology articles indexed using the Web of Science during 2002–2011**

	Keywords	Frequencies
1	forest	9302
2	diversity	5424
3	conservation	5135
4	dynamics	4886
5	vegetation	4720
6	biodiversity	4613
7	patterns	4166
8	growth	4069
9	rain-forest	3253
10	management	3236
11	nitrogen	3136
12	forests	3069
13	soil	2793
14	ecology	2677
15	communities	2596
16	carbon	2568
17	climate-change	2412
18	ecosystems	2407
19	disturbance	2389
20	species richness	2381
21	boreal forest	2334
22	landscape	2180
23	biomass	2130
24	model	2100
25	climate	2095
26	fire	2043
27	abundance	1855
28	united-states	1849
29	habitat	1846
30	temperature	1824
31	plants	1782
32	organic-matter	1755
33	populations	1733
34	decomposition	1603
35	climate change	1599
36	dispersal	1590
37	responses	1576
38	regeneration	1531
39	tropical forest	1513
40	land-use	1509
41	habitat fragmentation	1495
42	trees	1486
43	fragmentation	1473
44	forest soils	1441
45	evolution	1408
46	succession	1384
47	deforestation	1375
48	ecosystem	1362
49	birds	1333
50	population	1276
51	competition	1273
52	water	1235
53	variability	1210
54	deciduous forest	1190
55	forest management	1189
56	community structure	1178
57	behavior	1140
58	community	1131
59	restoration	1127
60	tropical forests	1107
61	photosynthesis	1093
62	seed dispersal	1081
63	usa	1067
64	productivity	1054
65	microbial biomass	1040
66	density	1034
67	impact	1019
68	brazil	1018
69	models	988
70	carbon-dioxide	978
71	phosphorus	971
72	size	971
73	predation	947
74	classification	943
75	respiration	932
76	scale	927
77	drought	920
78	national-park	918
79	plant	910
80	selection	909
81	tree	902
82	deposition	889
83	history	888
84	recruitment	875
85	norway spruce	874
86	soil respiration	870
87	australia	868
88	consequences	864
89	tropical rain-forest	839
90	survival	834
91	quality	830
92	mexico	819
93	costa-rica	813
94	impacts	812
95	new-zealand	796
96	forest soil	794
97	mortality	788
98	soils	787
99	grassland	786
100	assemblages	785

### Articles analysis result

#### By country/region

The 78,986 articles were analyzed by countries or regions and sorted in reverse order by their total numbers and Table 3 lists the results for the top 20 countries. We supplemented a column in the original table and classified these 20 countries/regions by their respective continents, which showed that North America and 12 European countries had about 44.71% and 42.35% of all the articles, respectively, indicating published articles related to forest ecology in North America and Europe predominate.

**Table 3:** **Top 20 countries/regions publishing articles on forest ecology indexed using the web of science during 2002–2011**

	Countries/Regions	Records	Ratio (%)	Continents
1	USA	28060	35.53	North America
2	Canada	7255	9.19	North America
3	Germany	6311	7.99	Europe
4	Brazil	4561	5.77	Africa
5	Australia	4375	5.54	Australia
6	England	4229	5.35	Europe
7	Peoples R China	4122	5.22	Asia
8	France	3930	4.98	Europe
9	Japan	3504	4.44	Asia
10	Spain	3402	4.31	Europe
11	Sweden	2708	3.43	Europe
12	Finland	2417	3.06	Europe
13	Italy	2230	2.82	Europe
14	Netherlands	1921	2.43	Europe
15	Switzerland	1871	2.37	Europe
16	India	1798	2.28	Asia
17	Mexico	1572	1.99	South America
18	Russia	1554	1.97	Europe
19	Scotland	1455	1.84	Europe
20	New Zealand	1421	1.80	Europe

The combined frequency of keywords related to tropical forest, represented by “rain-forest” (3,253), “tropical forest” (1,513), “tropical forests” (1,107), and “tropical rain-forest” (839), totaled 6,712 keyword entries, which was exceeded only by the keyword “forest” with 9,302 entries (Table 2). This indicates that tropical forest was the main focus of research in forest ecology studies. Tropical forest is mainly distributed in Southeast Asia, Central America, South America, Australia, Africa. However, the main countries with strong research capabilities related to tropical forest research were not located in those areas, but were found in North America and Europe.

#### By organization

Forest ecology studies were conducted by 7,598 organizations, and Table 4 lists the top 20 organizations and their related countries. The University of California System, the Chinese Academy of science, and US Forest Service produced the most articles. Eight organizations were from the USA, two each from Canada, Brazil, and Germany, and the remaining six were from China, Sweden, Finland, Russia, Spain, and France.

**Table 4:** **Top 20 organizations publishing articles on forest ecology indexed using the web of science during 2002–2011**

	Organizations	Records	Ratio (%)	Counties
1	Univ Calif System	2749	3.48	USA
2	Chinese Acad SCI	2359	2.99	China
3	US Forest Serv	2203	2.79	USA
4	Swedish Univ Agr SCI	1342	1.70	Sweden
5	Oregon State Univ	1200	1.52	USA
6	Univ Helsinki	1055	1.34	Finland
7	Univ British Columbia	1008	1.28	Canada
8	Univ Wisconsin System	978	1.24	USA
9	Univ Alberta	973	1.23	Canada
10	Russian Acad SCI	925	1.17	Russia
11	Univ Florida	905	1.15	USA
12	USDA	905	1.15	USA
13	Univ Sao Paulo	896	1.13	Brazil
14	US Geol Survey	883	1.12	USA
15	Univ Fed Santa Maria	868	1.10	Brazil
16	Smithsonian Inst	867	1.10	USA
17	Max Planck Society	808	1.02	Germany
18	Univ Gottingen	785	0.99	Germany
19	INRA	771	0.98	France
20	CSIC	766	0.97	Spain

*USDA* United States department of agriculture, *INRA* Institut National de la recherche agronomique, *CSIC* consejo superior de investigaciones científicas.

#### By funding agency

6,356 funding agencies subsidized forest ecology studies, and the top 20 were exported for closer analysis. Because many articles used abbreviations for the funding agencies the top 20 were merged into 15 (Table 5). Examples include the National Science Foundation (NSF), the Conselho Nacional de Desenvolvimento Científico e Tecnológico (CNPq), the European Union (EU), and the Natural Sciences and Engineering Research Council of Canada (NSERC).

**Table 5:** **The 15 most productive agencies funding forest ecology research indexed by the web of science during 2002–2011**

	Funding agencies	Articles number	Ratio (%)	Countries
1	National Science Foundation	2240	2.84	USA
2	National Natural Science Foundation of China	831	1.05	China
3	Natural Sciences and Engineering Research Council of Canada	807	1.02	Canada
4	CNPq	744	0.94	Brazil
5	European Union	601	0.76	EU
6	Chinese Academy of Sciences	372	0.47	China
7	NASA	357	0.45	USA
8	European Commission	337	0.43	EC
9	Academy of Finland	311	0.39	Finland
10	Australian Research Council	265	0.34	Australia
11	CAPES	221	0.28	Brazil
12	National Basic Research Program of China	196	0.25	China
13	FAPESP	192	0.24	Brazil
14	Russian Foundation for Basic Research	185	0.23	Russia
15	USDA Forest Service	172	0.22	USA

CNPQ: Conselho Nacional de Desenvolvimento Científico e Tecnológico, Brazil; NASA: National Aeronautics and Space Administration, USA; CAPES: Coordenação de Aperfeiçoamento de Pessoal de Nivel Superior, Brazil; FAPESP: Fundação de Amparo à Pesquisa do Estado de São Paulo, Brazil; EC: European Commission.

The National Science Foundation (USA), National Natural Science Foundation of China (China), Natural Sciences and Engineering Research Council of Canada (Canada), Conselho Nacional de Desenvolvimento Científico e Tecnológico (Brazil), and European Commission were more prolific in forest ecology than other funding agencies. Combining the number of articles in Table 5 by country/region demonstrates that the USA (2,769), China (1,399), Brazil (1,157), Canada (807), and EU (601) were also the top five countries/regions and provided more financial aid to forest ecology research than other countries.

#### By research area

In the analysis, forest ecology was related to 72 research areas identified by the Web of Science data. Table 6 lists the top 20 research areas and clearly shows that forest ecology studies were related to a wide range of disciplines. Environmental sciences ecology (31,172 or 39.47% of all articles), forestry (13,164, 16.67%), agriculture (8,354, 10.58%), and plant sciences (8,027, 10.16%) were the top four major related research areas.

**Table 6:** **The top 20 research areas related to forest ecology indexed using the web of science during 2002–2011**

	Research areas	Articles number	Ratio (%)
1	Environmental Sciences Ecology	31172	39.47
2	Forestry	13164	16.67
3	Agriculture	8354	10.58
4	Plant Sciences	8027	10.16
5	Zoology	6470	8.19
6	Biodiversity Conservation	6005	7.60
7	Geology	5660	7.17
8	Meteorology Atmospheric Sciences	3654	4.63
9	Physical Geography	3453	4.37
10	Water Resources	2521	3.19
11	Marine Freshwater Biology	2271	2.88
12	Entomology	2176	2.76
13	Engineering	1981	2.51
14	Life Sciences Biomedicine Other Topics	1650	2.09
15	Evolutionary Biology	1631	2.07
16	Remote Sensing	1611	2.04
17	Science Technology Other Topics	1319	1.67
18	Biochemistry Molecular Biology	1269	1.61
19	Imaging Science Photographic Technology	1205	1.53
20	Genetics Heredity	1079	1.37

#### By author

A total of 48,373 authors participated in forest ecology related studies. Among the 20 authors publishing the most articles, five were from the USA, four were from Canada, and two each were from Belgium, Finland, and England (Table 7).

**Table 7:** **The 20 most productive authors of research papers related to forest ecology indexed using the Web of Science during 2002–2011**

	Authors	Authors’ countries	Articles number	Ratio (%)
1	Bergeron Y	Canada	146	0.19
2	Kulmala M	Finland	123	0.16
3	Hermy M	Belgium	114	0.14
4	Lindenmayer DB	Australia	110	0.14
5	Black TA	Canada	103	0.13
6	Coops NC	Canada	95	0.12
7	Asner GP	USA	91	0.12
8	Verheyen K	Belgium	91	0.12
9	Reich PB	USA	87	0.11
10	Penuelas J	Spain	85	0.11
11	Vesala T	Finland	85	0.11
12	Leuschner C	Germany	81	0.10
13	Peres CA	England	81	0.10
14	Chen JM	Canada	80	0.10
15	Ciais P	France	80	0.10
16	Groffman PM	USA	79	0.10
17	Law BE	USA	78	0.10
18	Malhi Y	England	78	0.10
19	Fahey TJ	USA	77	0.10
20	Yu GR	China	77	0.10

#### By year

**Figure 2:**
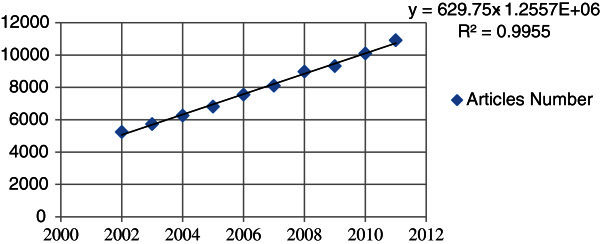
**A linear relationship between articles number and years during 2002-2011.**

From 2002 to 2011, the annual number of published articles about forest ecology was growing at a stable rate (Table 8), as the fit produced a high determination coefficient from the collected data (R^2^ = 0.9955). The best fit for forest ecology was found to be: *y* = 629.75*x* – 1.2557*exp* + 06, where *y* is the article number and *x* is the number of years since 2002. Extrapolating from the model, the number of articles about forest ecology in the following years could be forecasted (Figure 2).

**Table 8:** **Annual number of articles on forest ecology indexed using the Web of Science during 2002–2011**

	Years	Articles number	Ratio (%)
1	2002	5245	6.64
2	2003	5729	7.25
3	2004	6250	7.91
4	2005	6816	8.63
5	2006	7555	9.57
6	2007	8098	10.25
7	2008	8970	11.36
8	2009	9311	11.79
9	2010	10096	12.78
10	2011	10915	13.82

#### By publication

The number of journals publishing forest ecology related articles each year increased from 430 in 2002 to 856 in 2011. Table 9 shows the top 20 major journals indicating that *Forest Ecology and Management* (3,876, 4.91%) was the top journal on forest ecology by article count, followed by *Canadian Journal of Forest Research* (1,399, 1.77%) and *Biological Conservation* (1,399, 1.77%).

**Table 9:** **The top 20 journals related to forest ecology analyzed using the Web of Science during 2002–2011**

	Publications	Articles number	Ratio (%)
1	Forest Ecology and Management	3876	4.91
2	Canadian Journal of Forest Research	1399	1.77
3	Biological Conservation	933	1.18
4	Soil Biology Biochemistry	929	1.18
5	Biodiversity and Conservation	928	1.18
6	Global Change Biology	824	1.04
7	Ecology	750	0.95
8	Oecologia	741	0.94
9	Biotropica	666	0.84
10	Plant and Soil	653	0.83
11	Ecological Applications	636	0.81
12	Plant Ecology	614	0.78
13	Ecological Modeling	599	0.76
14	Remote Sensing of Environment	598	0.76
15	Argicultural and Forest Meteorology	589	0.75
16	Journal of Tropical Ecology	543	0.69
17	Journal of Geophysical Research Atmospheres	523	0.66
18	Conservation Biology	516	0.65
19	Journal of Biogeography	510	0.65
20	Tree Physiology	508	0.64

## Discussion

The results of this study pointed to several significant hotspots in global research related to forest ecology based on an analysis of article keywords for articles published during 2002–2011, and revealed the distribution of the articles from seven aspects listed above. The keyword analysis method and the java analysis program could be extended to other related research fields.

In the keywords analysis, we presumed that a keyword appeared only once in the keywords list of an article (Campbell 1963 ). Therefore the frequency of a keyword could show the number of articles that had used this keyword. For example, the frequency of “forest” was 9,302, meaning that 9,302 articles had used “forest” as a keyword in 73,740 articles.

It was undisputed that “forest” was the most frequently used keyword (9,302 articles). Most writers used this word to express the concept of “forest” instead of its plural “forests”; therefore, “forest” appeared in articles three times more than “forests” (3,069). The next four most frequently used words were “diversity” (5,424), “conservation” (5,135), “dynamics” (4,886), and “vegetation” (4,720) indicating forest diversity, forest conservation, forest dynamics and forest vegetation were the focus of forest ecological studies.

The frequency of “patterns” (4,166), “model” (2,100), and “models” (988) demonstrated that these words were widely used in forest developmental pattern and model studies. The keywords “management” (3,236), “ecology” (2,677), “ecosystems” (2,407), and “ecosystem” (1,362) were also frequently used in macro research (9,682 times), accounting for 1.03% in all keywords indicating large numbers of studies had been carried out in these aspects of forest research in last ten years.

USA” (2,916), “Brazil” (1,018), “Australia” (868), “Mexico” (819), “Costa Rica” (813) and “New Zealand” (796) appeared more frequently than the names of other countries showing that many studies focused on those countries. During the early twenty-first century, the warm droughts in the United States, Europe and Australia have been recognized as a considerable change from the climatological conditions and variability of the late twentieth century (Dai 2011 ), and the focus of forest ecology studies in those regions were impacted accordingly. From a regional point of view, we can see that the total frequencies of “rain-forest” (3,253), “tropical forests” (1,107), and “tropical forest” (1,513) were 5,873, 2.5 times more frequent than “boreal forest” (2,334), indicating that forest ecology studies concerning tropical forests were produced more frequently than those related to boreal forests.

In 2005, large-scale, warm droughts occurred in North America, Africa, Europe, Amazonia and Australia, resulting in major effects on terrestrial ecosystems, carbon balance and food security (Breshears 2005 ). The words “nitrogen” (3,136), “carbon” (2,568), and “phosphorus” (971) were used frequently in the studies concerning elemental nutrients. There were numerous studies related to how the climate is affecting forest ecology, as indicated by the frequencies of “climate-change”, “climate”, and “climate change,” which were 2,412, 2,095 and 1,599, respectively.

This study did reveal some problem areas. Some keywords were not being used consistently, such as soil, soils, forest soil and forest soils, which all pointed to the same thing: forest soil. Another example was that tropical forest and tropical forests also expressed similar meanings. The use of multiple keywords for a single concept might be related to the writing styles and habits of different authors, but this creates difficulty in statistical analysis.

The USA, Canada, and Germany were the top three most productive countries of forest ecology related research. The most three productive organizations were the University of California System, Chinese Academy of Sciences, and the US Forest Service. The three most productive funding agencies were the National Science Foundation, the National Natural Science Foundation of China, and the Natural Sciences and Engineering Research Council of Canada. Environmental science / ecology, forestry, and agriculture were the top three most popular categories. The spatial clusters of authors were mainly in the USA and Canada. *Forest Ecology and Management*, *Canadian Journal of Forest Research*, and *Biological Conservation* were the top three journals with the most publications related to forest ecology research. In the article analysis, the results by country/region, organization, funding agency, author distribution, and sources titles, was clustered in developed countries, apparently because these countries have economic strength required to invest in science and technology.

In this study, the limitations of search term expressions and the English language made it impossible to include all related keywords in the field of forest ecology research, especially in other languages. This study did not analyze the effects of cooperation between authors and joint papers by authors from multiple nations. In the journal sort, the impact factor of the journal was not considered.

## Conclusions

A serial java program was developed and applied to conduct keyword frequency analysis. That improved the efficiency of data processing and provided an analysis method. Keyword analysis offered insight into forest ecology research areas of interest, while the abundance of less frequent keywords suggested a lack of continuity in research and a wide disparity in the focus of forest ecology research. The top 100 keywords in the keyword analysis were almost all included in the top 20 research areas in the article analysis, so one could conclude that keyword frequency analysis is consistent with article research area analysis. Their difference is the former is concrete and the latter is abstract.Appendix A(TS = (habitats) or TS = (species diversity) or TS = (biodiversity) or TS = (species richness) or TS = (environmental factors) or TS = (ecosystems) or TS = (plant communities) or TS = (landscape) or TS = (phenology) or TS = (environmental degradation) or TS = (plant) or TS = (populations) or TS = (animal) or TS = (ecological disturbance) or TS = (landscape) or TS = (synecology) or TS = (palaeo ecology) or TS = (community) or TS = (biogeography) or TS = (population) or TS = (ecotypes) or TS = (predator prey relationships) or TS = (microbial) or TS = (freshwater) or TS = (food webs) or TS = (lowland areas) or TS = (restoration) or TS = (fire) or TS = (food chains) or TS = (autecology) or TS = (marine) or TS = (chemical) or TS = (human) or TS = (bioenergetics) or TS = (ecological balance) or TS = (bio coenosis) or TS = (microenvironments) or TS = (dendro ecology) or TS = (climate) or TS = (soils) or TS = (physiography) or TS = (carbon balance) or TS = (nutrient cycling) and (TS = (forest).

## Electronic supplementary material

Additional file 1: **Appendix B: count.java, Appendix C: merge.java, Appendix D: makejar.bat, Appendix E: count.bat, and Appendix F:merge.bat.** (DOCX 22 KB)
