# Supplementary material for: A bibliometric analysis of global forest ecology research during 2002–2011
Source: Springerplus. 2013 May 2;2:204. doi: 10.1186/2193-1801-2-204 (PMC3653034; doi:10.1186/2193-1801-2-204)
Supplement: Supplementary file 1 — Additional file 1: Appendix B: count.java, Appendix C: merge.java, Appendix D: makejar.bat, Appendix E: count.bat, and Appendix F:merge.bat. (DOCX 22 KB) [file 40064_2013_258_MOESM1_ESM.docx]

**Appendix B: count.java**

import java.io.*;

import static java.lang.Math.*;

import static java.math.BigInteger.*;

import static java.util.Arrays.*;

import static java.util.Collections.*;

import java.math.*;

import java.util.*;

class count {

static HashMap<String,Integer>hm=new HashMap<String, Integer>();

public static void main(String[] args) throws IOException {

String name="";

String[]start;

String cont="";

if(args.length==3){

start=args[0].split("`");

cont=args[1];

name=args[2];

}

else return;

FileInputStream fis=new FileInputStream(name);

InputStreamReader isr=new InputStreamReader(fis,"UTF-8");

FileOutputStream fos1=new FileOutputStream("[filter]"+name);

OutputStreamWriter osw1=new OutputStreamWriter(fos1,"UTF-8");

FileOutputStream fos2=new FileOutputStream("[times]"+name);

OutputStreamWriter osw2=new OutputStreamWriter(fos2,"UTF-8");

//f = new BufferedReader(new FileReader(front+name));

//out = new PrintWriter(new BufferedWriter(new FileWriter(front+"[filter]"+name)));

//out2 = new PrintWriter(new BufferedWriter(new FileWriter(front+"[times]"+name)));

f = new BufferedReader(isr);

out = new PrintWriter(new BufferedWriter(osw1));

out2 = new PrintWriter(new BufferedWriter(osw2));

boolean in=false;

String cur="";

while(true){

String s=nl();

if(s==null)break;

boolean st=false;

String which="";

for (int i = 0; i < start.length; i++) {

if(s.startsWith(start[i])){

st=true;

which=start[i];

}

}

if(st){

if(cur.length()>0){

doit(cur);

}

cur="";

out.println(s.substring(which.length()));

cur+=s.substring(which.length())+" ";

in=true;

}

else if(in&&s.startsWith(cont)){

cur+=s.substring(cont.length())+" ";

out.println(s);

}

else{

in=false;

if(cur.length()>0){

doit(cur);

}

cur="";

}

}

if(cur.length()>0){

doit(cur);

}

Comp[]res = new Comp[hm.size()];

int resl=0;

for (Map.Entry<String,Integer> entry : hm.entrySet()) {

res[resl++]=new Comp(entry.getKey(),entry.getValue());

}

sort(res);

for (int i = 0; i < resl; i++) {

out2.println(res[i].str+":"+res[i].cnt);

}

out2.close();

out.close();

System.exit(0);

}

static public class Comp implements Comparable<Comp> {

public int compareTo(Comp other) {// myself first asc

if(cnt==other.cnt)

return str.compareTo(other.str);

return other.cnt-cnt;

}

String str;int cnt;

public Comp(String str, int cnt) {

super();

this.str = str;

this.cnt = cnt;

}

}

private static void doit(String cur) {

String[]sp=cur.split(";");

for (int i = 0; i < sp.length; i++) {

String x=sp[i].trim().toLowerCase();

if(hm.containsKey(x))hm.put(x, hm.get(x)+1);

else hm.put(x, 1);

}

}

static PrintWriter out;

static PrintWriter out2;

static BufferedReader f;

static StringTokenizer st;

static String nl() throws IOException {

try {

return f.readLine();

} catch (Exception e) {

return null;

}

}

static int inf = Integer.MAX_VALUE / 2;

static boolean in(int a, int l, int h) {

return l <= h ? a >= l && a <= h : in(a, h, l);

}

static int ip(String s) {

return Integer.parseInt(s);

}

static long lp(String s) {

return Long.parseLong(s);

}

static int gcd(int a, int b) {

return b == 0 ? a : gcd(b, a % b);

}

static String mins(String a, String b) {

return a.compareTo(b) < 0 ? a : b;

}

static void filli(Object array, int val) {

if (array instanceof int[])

Arrays.fill((int[]) array, val);

else

for (Object o : (Object[]) array)

filli(o, val);

}

static boolean isPrime(int number) {

if (number < 2)

return false;

if (number == 2)

return true;

if (number % 2 == 0)

return false;

for (int i = 3; i * i <= number; i += 2)

if (number % i == 0)

return false;

return true;

}

}

**Appendix C: merge.java**

import java.io.*;

import static java.lang.Math.*;

import static java.math.BigInteger.*;

import static java.util.Arrays.*;

import static java.util.Collections.*;

import java.math.*;

import java.util.*;

class merge {

static HashMap<String,Integer>hm=new HashMap<String, Integer>();

public static void main(String[] args) throws IOException {

FileOutputStream fos1=new FileOutputStream("["+args.length+"]mergetimes.txt");

OutputStreamWriter osw1=new OutputStreamWriter(fos1,"UTF-8");

out = new PrintWriter(new BufferedWriter(osw1));

for (int i = 0; i < args.length; i++) {

FileInputStream fis=new FileInputStream(args[i]);

InputStreamReader isr=new InputStreamReader(fis,"UTF-8");

f = new BufferedReader(isr);

while(true){

String s=nl();

if(s==null)break;

String[]sp=s.split(":");

if(sp.length!=2)continue;

if(hm.containsKey(sp[0]))hm.put(sp[0], hm.get(sp[0])+ip(sp[1]));

else hm.put(sp[0], ip(sp[1]));

}

f.close();

}

Comp[]res = new Comp[hm.size()];

int resl=0;

for (Map.Entry<String,Integer> entry : hm.entrySet()) {

res[resl++]=new Comp(entry.getKey(),entry.getValue());

}

sort(res);

for (int i = 0; i < resl; i++) {

out.println(res[i].str+":"+res[i].cnt);

}

out.close();

System.exit(0);

}

static public class Comp implements Comparable<Comp> {

public int compareTo(Comp other) {// myself first asc

if(cnt==other.cnt)

return str.compareTo(other.str);

return other.cnt-cnt;

}

String str;int cnt;

public Comp(String str, int cnt) {

super();

this.str = str;

this.cnt = cnt;

}

}

static PrintWriter out;

static BufferedReader f;

static StringTokenizer st;

static String nl() throws IOException {

try {

return f.readLine();

} catch (Exception e) {

return null;

}

}

static String nt() throws IOException {

try {

if (st == null || st.hasMoreTokens() == false) {

st = new StringTokenizer(f.readLine());

}

return st.nextToken();

} catch (Exception e) {

return null;

}

}

static int inf = Integer.MAX_VALUE / 2;

static boolean in(int a, int l, int h) {

return l <= h ? a >= l && a <= h : in(a, h, l);

}

static int ip(String s) {

return Integer.parseInt(s);

}

static long lp(String s) {

return Long.parseLong(s);

}

static int gcd(int a, int b) {

return b == 0 ? a : gcd(b, a % b);

}

static String mins(String a, String b) {

return a.compareTo(b) < 0 ? a : b;

}

static void filli(Object array, int val) {

if (array instanceof int[])

Arrays.fill((int[]) array, val);

else

for (Object o : (Object[]) array)

filli(o, val);

}

static boolean isPrime(int number) {

if (number < 2)

return false;

if (number == 2)

return true;

if (number % 2 == 0)

return false;

for (int i = 3; i * i <= number; i += 2)

if (number % i == 0)

return false;

return true;

}

}

**Appendix D: makejar.bat**

del *.class frequency.jar

"C:\Program Files\Java\jre7\bin\javac" count.java merge.java

"C:\Program Files\Java\jre7\bin\jar" cvf frequency.jar *.class

del *.class

**Appendix E: count.bat**

"C:\Program Files\Java\jre7\bin\java" -cp .;frequency.jar count "DE `ID " " " "1.txt"

"C:\Program Files\Java\jre7\bin\java" -cp .;frequency.jar count "DE `ID " " " "2.txt"

"C:\Program Files\Java\jre7\bin\java" -cp .;frequency.jar count "DE `ID " " " "3.txt"

……………..

"C:\Program Files\Java\jre7\bin\java" -cp .;frequency.jar count "DE `ID " " " "156.txt"

"C:\Program Files\Java\jre7\bin\java" -cp .;frequency.jar count "DE `ID " " " "157.txt"

"C:\Program Files\Java\jre7\bin\java" -cp .;frequency.jar count "DE `ID " " " "158.txt"

**Appendix F: merge.bat**

"C:\Program Files\Java\jre7\bin\java" -cp .;frequency.jar merge "[times]1.txt" "[times]2.txt" "[times]3.txt" … "[times]157.txt" "[times]158.txt"
